# Antimalarial Effect of the Total Glycosides of the Medicinal Plant, *Ranunculus japonicus*

**DOI:** 10.3390/pathogens10050532

**Published:** 2021-04-28

**Authors:** Hae-Soo Yun, Sylvatrie-Danne Dinzouna-Boutamba, Sanghyun Lee, Zin Moon, Dongmi Kwak, Man-Hee Rhee, Dong-Il Chung, Yeonchul Hong, Youn-Kyoung Goo

**Affiliations:** 1 Department of Parasitology and Tropical Medicine, School of Medicine, Kyungpook National University, Daegu 41944, Korea; sooandart@naver.com (H.-S.Y.); sylvatriez@yahoo.fr (S.-D.D.-B.); zinmoonkcn@gmail.com (Z.M.); dichung@knu.ac.kr (D.-I.C.); ychong@knu.ac.kr (Y.H.); 2 Division of Zoonotic and Vector Borne Disease Research, Center for Infectious Diseases, Korea National Institute of Health, Korea CDC, Chungbuk 28159, Korea; cdcsanghyun@gmail.com; 3 Department of Microbiology, College of Medicine and Medical Research Institute, Chungbuk National University, Cheongju 28644, Korea; 4 Laboratory of Parasitology, College of Veterinary Medicine, Kyungpook National University, Daegu 41566, Korea; dmkwak@knu.ac.kr; 5 Laboratory of Veterinary Physiology and Cell Signaling, College of Veterinary Medicine, Kyungpook National University, Daegu 41566, Korea; rheemh@knu.ac.kr

**Keywords:** *Ranunculus japonicus*, plasmodium falciparum, plasmodium berghei, antimalarial effect

## Abstract

In traditional Chinese medicine, *Ranunculus japonicus* has been used to treat various diseases, including malaria, and the young stem of *R. japonicus* is consumed as a food in the Republic of Korea. However, experimental evidence of the antimalarial effect of *R. japonicus* has not been evaluated. Therefore, the antimalarial activity of the extract of the young stem of *R. japonicus* was evaluated in vitro using both chloroquine-sensitive (3D7) and chloroquine-resistant (Dd2) strains; in vivo activity was evaluated in *Plasmodium berghei*-infected mice via oral administration followed by a four-day suppressive test focused on biochemical and hematological parameters. Exposure to extracts of *R. japonicus* resulted in significant inhibition of both chloroquine-sensitive (3D7) and resistant (Dd2) strains of *P. falciparum,* with IC_50_ values of 6.29 ± 2.78 and 5.36 ± 4.93 μg/mL, respectively. Administration of *R. japonicus* also resulted in potent antimalarial activity against *P. berghei* in infected mice with no associated toxicity; treatment also resulted in improved hepatic, renal, and hematologic parameters. These results demonstrate the antimalarial effects of *R. japonicus* both in vitro and in vivo with no apparent toxicity.

## 1. Introduction

*Ranunculus japonicus* is of the tribe *Ranunculeae* and the family *Ranunculaceae*. In China and the Republic of Korea, *R. japonicus* has been used to treat malaria, jaundice, migraines, stomachaches, arthralgia, crane-like arthropathy, ulcers, toothaches, and eye inflammation [1]. The young stem of *R. japonicus* is known as a food in the Republic of Korea. In addition, the total glycosides of *R. japonicus* showed anti-inflammatory, analgesic, and anti-angiotensin II effects [2,3]. However, experimental evidence of the antimalarial effect of *R. japonicus* has not been evaluated.

Malaria is among the most serious of vector-borne infections; the disease ensues upon transfer of *Plasmodium* sporozoites from infected *Anopheles* mosquitoes to human targets [4]. According to the World Health Organization (WHO), half of the world’s population is currently at risk for malaria; in 2019, 229 million cases of malaria were reported, with the death toll reaching 409,000 [5]. Malaria remains a major challenge for healthcare providers in the developing world; 81% of the most recent data on malaria cases were from Africa, followed by the regions of Southeast Asia (13%) and the Eastern Mediterranean (5%) [6].

Although malaria is a life-threatening disease, it can be controlled with early diagnosis and prompt treatment [7,8]. Among the available medications, chloroquine (CQ) was synthesized from quinoline and became the most widely used antimalarial drug by the 1940s due to its low cost and wide range of efficacy [9,10]. However, resistance to this drug increased at a rapid rate in Asia, Latin America, and Africa [11,12]. Now, artemisinin- and artemisinin-based combination therapy are used as the first-line treatment for all cases of malaria, especially in regions where *Plasmodium falciparum* infections predominate [13]. However, the WHO has reported confirmed cases of artemisinin resistance in five countries in the Greater Mekong Subregion, including Cambodia, the Lao People’s Democratic Republic, Myanmar, Thailand, and Viet Nam [14]. Of particular concern, strains of *P. falciparum* isolated from cases of malaria identified at the Cambodia–Thailand border are comparatively resistant to all known effective anti-malarials; as such, there is a real and substantial risk that multidrug resistance will emerge at other locations as well.

As has become clear, antimalarial drug resistance to *P*. *falciparum* is an ongoing and increasing problem. In addition to increases in morbidity and mortality, drug resistance has resulted in increased associated costs due to the ongoing efforts to improve the efficacy of antimalarial treatments, which indicates an urgent need for the discovery and development of new antimalarial agents. Medicinal food approaches are clearly feasible and have been beneficial with respect to drug discovery for malaria [15]. As such, we evaluated the antimalarial activity of an extract from the young stem of *Ranunculus japonicus* (RJE) and the parameters indicating physiopathological changes induced by *P. berghei* and RJE treatment to reveal the status of infection and therapeutic efficacy of RJE.

## 2. Results

### 2.1. Antimalarial Impact and Cytotoxicity of the Ranunculus japonicus Extract in Studies Performed In Vitro

As any novel antimalarial drug needs to be effective against both CQ-sensitive and -resistant strains of *P. falciparum*, the impact of the extract prepared from *R. japonicus* was evaluated in an in vitro culture of red blood cells (RBCs) infected with both 3D7 and Dd2 strains. We found that the RJE inhibited parasite growth in a dose-dependent manner; the calculated IC_50_ values of the 3D7 and Dd2 strains were 6.29 ± 2.78 and 5.36 ± 4.93 μg/mL, respectively. The RJE was also tested for potential cytotoxicity against human foreskin fibroblasts (HFFs); the results indicated very low toxicity with high selectivity indices (138.48 for 3D7 and 157.82 for Dd2; Table 1).

### 2.2. Antimalarial Impact of the R. japonicus Extract Used to Treat Mice Infected with Plasmodium berghei

**Figure 1:**
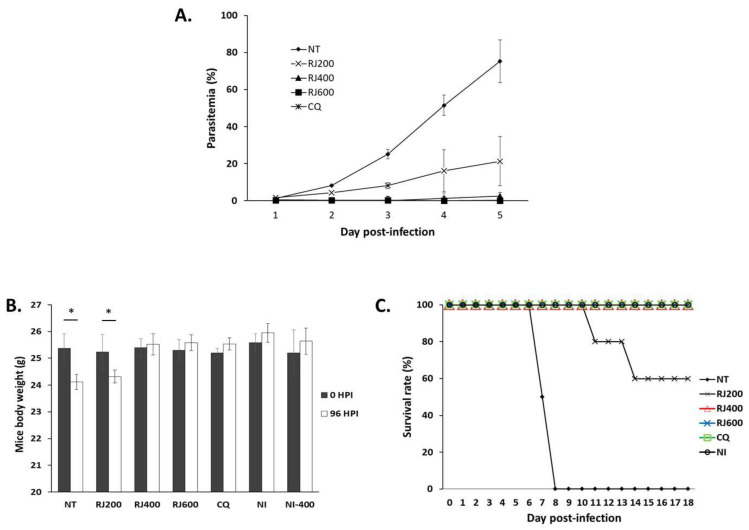
Antimalarial impact of *R. japonicus* extract on parasitemia, total body weight, and survival of *P. berghei*-infected mice. (**A**) An extract of *R. japonicus* was administered orally to *P. berghei*-infected mice on four successive days after inoculation; parasitemia was evaluated daily. (**B**) Total body weights were measured at day 0 and at the end of drug administration for both the NT and five experimental groups. * *p* < 0.05 when comparing total body weights at 0 to 96 h post-inoculation in each of the groups evaluated. (**C**) Survival was evaluated daily in all groups of mice.

Because *P. falciparum* cannot infect mice, the rodent malarial pathogen, *P. berghei*, was used to evaluate the impact of the RJE in vivo. Parasitemia, body weight, and survival were monitored on days 0–4 after intraperitoneal inoculation with *P. berghei*-infected RBCs. Dose-dependent inhibition of parasite growth was observed in the groups of mice that were treated with 200, 400, or 600 mg/kg body weight/day of RJE (RJ200, RJ400, and RJ600 groups) at 96 h post-inoculation (Figure 1A). Specifically, 72.45%, 97.50%, and 98.14% inhibition were observed in RJ200, RJ400, and RJ600, respectively, compared to the level of parasitemia observed among *P. berghei*-infected mice that did not undergo treatment (NT). The efficacy of RJ400 and RJ600 was comparable with that observed in response to CQ, in which no parasites were detected. Similarly, groups of *P. berghei*-infected mice treated with RJE at 400 or 600 mg/kg/day gained an average of 1.88% and 2.46% of their original body weight at 96 h post-inoculation (Figure 1B). Increased body weight was also observed in those that had not been infected (NI), the NI400 and CQ groups, but significant reductions were observed among the mice of groups NT and RJ200 (4.71% and 3.23% decreases in bodyweight, respectively) at 96 h post-inoculation. Finally, we found that all mice of RJ400, RJ600, CQ, and NI survived 18 days post-inoculation. By contrast, all mice in the NT control group had died of malaria at day 8 post-inoculation. RJ200 had a 60% survival rate through day 18 post-inoculation (Figure 1C).

### 2.3. Impact of R. japonicus Extract on Hepatic Function of P. berghei-Infected Mice

As shown in Table 2, high levels of serum aspartate aminotransferase (AST) were detected in the NT group compared to the levels detected for the NI group. On the other hand, significant reductions in serum AST levels were detected in a comparison between mice in the NT group and the experimental groups, except for RJ200. Serum AST levels from mice of RJ400, RJ600, and CQ decreased by 19.6%, 20.3%, and 20.8%, respectively, compared to that of the NT mice. Moreover, serum alanine aminotransferase (ALT) levels were significantly different when comparing the results of the NT to the NI group. Significant reductions in serum ALT levels by 20.6%, 29.5%, 29.2%, and 35.4% were observed for the RJ200, RJ400, RJ600, and CQ groups, respectively, compared to that of the NT controls.

Serum levels of total bilirubin and indirect bilirubin were significantly higher in the NT group compared to the levels determined for the NI group. The treated groups revealed 42.5%, 46.1%, and 44.4% declines in total bilirubin in RJ400, RJ600, and CQ, respectively, and 52.6%, 76.7%, 76.3%, and 78.0% declines in indirect bilirubin in all groups treated with RJE or CQ, respectively. With respect to levels of direct bilirubin, the levels detected among mice in the NT group were greatly increased compared to those detected for the NI and experimental groups; the levels observed in response to treatment with 200, 400, or 600 mg/kg/day of the RJE and in response to CQ were more than two- to four-fold higher than those observed for the NT group.

### 2.4. Impact of the R. japonicus Extract on Renal Function of P. berghei-Infected Mice

Serum urea and creatinine levels differed significantly when comparing the NT group with the NI group (Table 3). Serum urea levels were significantly reduced by 42.0%, 51.8%, 51.3%, and 51.9% in the RJ200, RJ400, RJ600, and CQ groups, respectively, compared to levels detected in the NT group. Serum creatinine levels were significantly reduced in all treatment groups to 51.9%, 65.4%, 65.9%, and 67.4% of the levels detected in the NT, RJ200, RJ400, RJ600, and CQ groups, respectively.

### 2.5. Impact of R. japonicus Extract on Hematologic Parameters of P. berghei-Infected Mice

The erythrocyte count fell from 9.29 × 10^6^/µL in NI to 3.19 × 10^6^/µL in the NT group; the erythrocyte count was significantly higher in the RJ400 and RJ600 groups compared to in the NT group (Table 4). Likewise, the hematocrits of the NT group were reduced by 40.4% compared to those of the NI. Interestingly, the hematocrits of RJ400 and RJ600 mice were comparable to those of the NI, and the hematocrit values were even higher among those treated with CQ. Hemoglobin concentrations were also reduced by 49.2% in the NT group compared to the values observed for the NI group. By contrast, hemoglobin concentrations in all mice of RJ400 and RJ600 were comparable to those of the NI group. Likewise, reduced numbers of platelets were observed only in the NT group compared to the NI group, but not in the RJ400 or RJ600 group. By contrast, we detected high platelet counts in the CQ group at 104.1% of the levels observed for the NI group. Finally, the numbers of total leukocytes, lymphocytes, monocytes, and segmented neutrophils increased in response to infection (NT) (Table 5). Counts in all treated groups except RJ200 were comparable to those of the NI group.

## 3. Discussion

In a study, Carvalho et al. reported that an extract might be considered as having activity against malaria if one observes a more than 30% reduction in parasitemia in response to the treatment [16]. As such, our study suggests that the pharmacological effects of *R. japonicus* are high; treatment with this extract reduced *P. falciparum*-associated parasitemia. As shown in Table 1, ethanolic RJE inhibits the growth of both CQ-sensitive and CQ-resistant strains of *P. falciparum*. Strong inhibition of the *P. falciparum* Dd2 strain was observed compared with the response of the 3D7 strain; this result suggested that the mechanisms of action associated with this extract are different from those of the previously developed antimalarial agents. Earlier reports suggested that the antiplasmodial activity of natural extracts could be classified as follows: IC_50_ < 0.1 μg/mL, very good activity; IC_50_ between 0.1 and 1 μg/mL, good activity; IC_50_ between 1.1 and 10 μg/mL, good to moderate activity; IC_50_ between 11 and 50 μg/mL, low activity; IC_50_ > 100 μg/mL, inactive [17]. From our data, with an IC_50_ for strain 3D7 at 6.29 ± 2.78 μg/mL and for Dd2 at 5.36 ± 4.93 μg/mL, the RJE exhibits good to moderate activity against these target pathogens. Equally importantly, the extract promoted little to no cytotoxicity against human cells (Table 1), and the RBCs underwent no morphological changes when examined in vitro. Using a definition of SI > 10 as comparatively unharmful, the SIs associated with the RJE (>45 and >70, respectively) revealed little to no toxicity to cells in culture [18].

To evaluate the antimalarial impact of *R. japonicus* in an animal model, in vivo experiments were conducted using the *P. berghei* ANKA strain to infect wild-type mice. Using this model, the extract from *R. japonicus* showed significantly suppressed parasitemia at four days post-inoculation (96 h post-infection; *p* < 0.05) at rates from 72.45% to 98.14% compared to the NT; these findings validate the ethnomedical approach toward malaria management. However, the *R. japonicus*-treated mice group, especially when used at 200 mg/kg/day, resulted in weight loss at 96 h post-inoculation; increased body weight over this same period was observed in NI, CQ, and higher doses of *R. japonicus*. This decrease in body weight paralleled the survival rates; 60% of mice treated with 200 mg/kg/day of *R. japonicus* died by day 14 post-inoculation. These results suggested that the antimalarial impact of RJE was not as long-lasting or as effective as CQ in infected animal hosts. These low survival rates might also be due to the reemergence of *P. berghei* parasites after their initial clearance. Similar results with respect to mean survival time have been previously reported [19].

Malaria infection results in several pathophysiological changes due to the complex interactions that occur between the parasite and host. As such, we evaluated the parameters associated with hepatic, renal, and hematological responses to infection to gain a complete understanding of the therapeutic efficacy [20,21]. Treatment with RJE at doses at or greater than 400 mg/kg/day resulted in significant reductions in parasitemia; we found that parasite density was inversely associated with intact hepatic and renal functions. In *P. berghei* and *P. falciparum* infections, severe hepatic damage related to hepatic vessel congestion, periportal infiltration, and sinusoidal dilatation promoted direct changes in hepatic systems, increased the risk of liver failure, and induced metabolic modifications at glycemic levels [22]. The analyses of AST and ALT enzymatic activity and bilirubin concentrations are important markers of hepatic lesions in malaria. Reduced serum levels of AST, ALT, total bilirubin, and indirect bilirubin observed in response to treatment suggest that *R. japonicus* could protect *P. berghei*-infected animals against liver damage, thus improving their clinical status. Moreover, malaria infection also results in renal damage due to alteration of renal blood circulation caused by conformational changes of parasitized erythrocytes, increased blood viscosity, and cytoadherence of infected erythrocytes [23]. The observed decreases in serum levels of urea and creatinine suggest that reductions in parasitemia in response to administration of the RJE may also prevent erythrocyte adherence, high blood viscosity, dysfunctional renal blood circulation, and subsequent renal injury.

Hematological parameters, including RBC count, hemoglobin, hematocrit, platelet counts, and total white blood cell count, are frequently monitored as indicators of drug efficacy in malaria infection settings [24]. Parameters including the RBC count, hemoglobin, and hematocrit are all associated with anemia, which is the most common complication of malaria [25]. Hemoglobin functions in oxygen transport to tissues and promotes the oxidation of ingested food for energy release; it also transports carbon dioxide out of the body. Therefore, the hematocrit is an indicator of the ability to transport oxygen and absorb nutrients [26]. RJE treatment led to the restoration of diminished hemoglobin, hematocrit, and RBC counts observed in *P. berghei*-infected mice. Similarly, we observed an increase in the number of total leukocytes in the peripheral blood in response to infection; these results suggested that the host immune response was functioning in an attempt to combat the parasite [27]. The RJE treatment resulted in a significant reduction in the number of leukocytes recruited in response to infection with *P. berghei*; this may be a direct consequence of the *R. japonicus*-mediated reduction in parasite numbers.

Given its capacity to limit the growth of *Plasmodium* parasites in vitro and in vivo, RJE remains worthy of further evaluation. Notably, the discovery of the critical drug, artemisinin, began with the observation that an extract of *Artemisia annua L.* (Qinghao) resulted in only 12% to 40% growth inhibition in vitro [28].

## 4. Materials and Methods

### 4.1. Preparation of Herb Extracts

The RJE was prepared by following a previously described method, but with modifications [29]. Briefly, *R. japonicus* was collected from the Republic of Korea (GPS coordinates, 36°6′ N 127°28’ E). The air-dried and cut *R. japonicus* was extracted under reflex with 85% ethanol three times by filtration, pooling, and evaporation. After re-suspension in distilled water and partitioning in a 1:1 ration with ethyl acetate, the water fraction was eluted with water and ethanol by a D-101 macroporous resin column. The ethanol fraction formed the total glucosides of *R. japonicus*. Subsequently, the ethanol fraction was incubated in a rotary evaporator until all of the ethanol had evaporated. The dried extract solubilized in the culture medium or phosphate-buffered saline before experimental use.

### 4.2. Cell Cytotoxicity Assay

Cytotoxicity was evaluated in HFF cultures. Primary HFFs were maintained in Dulbecco’s modified Eagle’s medium containing 10% fetal calf serum, 100 units/mL penicillin/streptomycin, and 2 mM l-glutamine. To perform the cytotoxicity assay, cells were seeded in 96-well flat-bottom tissue culture clusters (Merck, Kenilworth, NJ, USA) at 10^4^ cells/well and incubated overnight. Cells were then treated with serial dilutions of the test compounds (final concentrations ranging from 10 to 1000 µg/mL), and cell proliferation was evaluated with the MTT (3-[4,5-dimethylthiazol-2-yl]-2,5-diphenyltetrazolium bromide, Sigma, St. Louis, MO, USA) assay after 72 h at 37 °C in 5% CO_2_ [30]. The results are expressed as the half-maximal drug inhibition concentration (IC_50_), which is the dose of compound necessary to inhibit cell growth by 50%. Three independent experiments were performed, with each trial performed in triplicate.

### 4.3. In Vitro Testing Against Plasmodium falciparum 3D7 and Dd2 Strains

The culture medium for this experiment included RPMI 1640 medium powder (Merck, USA), 10% normal human serum (A blood type, obtained from the Korean Red Cross), and 10 μg/mL gentamicin (Invitrogen, Carlsbad, CA, USA). Human RBCs to be infected and *P. falciparum* strains 3D7 and Dd2 were obtained from the Korean Red Cross and the Malaria Research and Reference Reagent Resource Center (MR4), respectively. Malarial strains were seeded with the medium and RBCs in cell culture plates and then incubated in 90% N_2_, 5% O_2_, and 5% CO_2_ until 2% parasitemia was reached. Cells were synchronized two times with 5% d-sorbitol (Sigma, Saint Louis, MO, USA) until only rings had formed prior to addition of the RJE. Concentrations of RJE at 100, 75, 50, 25, 10, and 1 μg/mL and CQ at 0.001, 0.01, 0.1, 1, and 10 μg/mL were prepared. In the case of the *P. falciparum* Dd2 strain, 0.001, 0.01, 0.1, 1, and 10 μg/mL artemisinin was included in the prepared culture. RJEs were added to cultures of synchronized *P. falciparum* parasite-infected RBCs (100 μL to each well in a 96-well plate). After 72 h, parasitemia was evaluated by light microscopy; IC_50_ values were calculated using Microsoft Excel. Each concentration was evaluated in triplicate and in three independent trials.

### 4.4. Selective Index

The selective index (SI) is a value that provides a measure of a substance’s toxicity to normal human cells compared to its toxicity against a parasite. SIs were calculated as the ratios between the IC_50_ values for cytotoxicity against HFFs and those calculated for *P. falciparum* strains 3D7 or Dd2.

### 4.5. In Vivo Testing Against the Plasmodium berghei ANKA Strain in Infected Mice

The antimalarial efficacy of the RJE was evaluated using a four-day suppressive test, as previously described [31]. Thirty-six female ICR mice (6 weeks old, weighing 25 ± 2 g) were purchased from Dae Han Bio Link Corporation, Eumsung, South Korea. Mice were randomly assigned into one of six groups, with six mice per group (NT: no treatment after *P. berghei* infection; RJ200, RJ400, and RJ600: 200, 400, and 600 mg/kg of RJE treatment after *P. berghei* infection, respectively; CQ: 10 mg/kg of CQ treatment after *P. berghei* infection; NI: no treatment and no infection; NI400: 400 mg/kg of RJE treatment without infection). For *P. berghei* infection, a donor mouse was infected with a frozen stock of *P. berghei* and then bled by cardiac puncture. Subsequently, mice that were prepared for inhibition assays were inoculated with 1 × 10^6^ *P. berghei* (ANKA strain)-infected mouse RBCs prepared from the donor mouse by intraperitoneal injection. These mice were then provided with oral doses of RJE (200, 400, and 600 mg/kg) or CQ by gavage; one group was maintained as an NT. Treatment was performed daily for a total of four days. Giemsa-stained smears were prepared from tail-vein blood sampled on days 0 through 4 (i.e., 96 h post-i.p. inoculation), and were examined under a microscope for quantitative evaluation of parasitemia. The growth inhibition of parasites was evaluated through a comparison with the no-treatment control group and was calculated as follows: Parasite inhibition rates = 100 − ([parasitemia determined in test group/parasitemia determined in NT group] × 100). Body weights and mortality were monitored daily. Experimental monitoring was terminated at 30 days post-infection. All animal experiments were approved by Kyungpook National University Animal Experiment Ethics Committee (Approval No. 2014–0082).

### 4.6. Blood Sample Preparation

For analysis of liver and kidney toxicity, as well as hematological parameters, 100–150 µL of blood was collected from the lateral tail vein of the tested mice in tubes with ethylenediaminetetraacetic acid (EDTA) after three days of treatment. Plasma samples for liver and kidney toxicity were prepared under centrifugation for 10 min (2000× *g*). Whole blood samples for hematological parameter analysis were stored at room temperature, and measurements were performed within 8 h after blood collection.

### 4.7. Liver and Kidney Toxicity

To evaluate the impact of RJE on hepatic function, the serum levels of aspartate aminotransferase (AST), alanine aminotransferase (ALT), and bilirubin were determined. The tests of renal function included serum levels of uric acid and creatinine. These values were determined using commercial enzyme-linked immunosorbent assay kits according to the manual provided by the company (Cloud-Clone Corp., Wuhan, China) [32].

### 4.8. Hematological Parameters

Complete blood counts were performed after three days of treatment using an automatic hematology analyzer (Celltac, Alpha VET MEK-6550, Nihon Kohden Co, Tokyo, Japan) with Data Management Software (DMS-Lite); these evaluations included erythrocytes, hemoglobin, hematocrit, platelet count, absolute number of leukocytes, lymphocytes, monocytes, and segmented neutrophils.

### 4.9. Statistical Analysis

The results were presented as means ± standard deviation. The data were analyzed using ANOVA followed by Tukey’s test (GraphPad Prism, San Diego, CA, USA), where *p* levels of significance ≤ 0.05, expressed as the mean standard deviation.

## Figures and Tables

**Table 1:** Antimalarial and cytotoxic activities of *Ranunculus japonicus* in *Plasmodium falciparum* 3D7 (chloroquine-sensitive) and Dd2 (chloroquine-resistant) strains.

Compound	IC_50_ in *Pf*3D7 (μg/mL)	IC_50_ in *Pf*Dd2 (μg/mL)	Cytotoxicity in HFF (μg/mL)	SI (HFF/*Pf*3D7)	SI (HFF/*Pf*Dd2)
*Ranunculus japonicus*	6.29 ± 2.78	5.36 ± 4.93	845.89 ± 13.28	134.48	157.82
Chloroquine	0.005 ± 0.001	0.40 ± 0.02	>45		
Artemisinin		0.002 ± 0.0009	>70		

IC_50_, the half-maximal drug inhibition concentration; *Pf*3D7, *Plasmodium falciparum* chloroquine-sensitive strain; *Pf*Dd2, *Plasmodium falciparum* chloroquine-resistant strain; HFF, human foreskin fibroblast; SI, selective index.

**Table 2:** Mean values ± standard deviations of hepatic function parameters in test groups.

	AST (U/L)	ALT (U/L)	Total Bilirubin (mg/dL)	Indirect Bilirubin (mg/dL)	Direct Bilirubin (mg/dL)
NT	61.28 ± 2.70	35.89 ± 2.81	1.42 ± 0.48	0.78 ± 0.23	0.19 ± 0.02
RJ200	54.42 ± 3.12	28.56 ± 2.85	0.91 ± 0.05	0.31 ± 0.09 *	0.42 ± 0.08
RJ400	50.12 ± 3.77 *	25.23 ± 1.91 *	0.82 ± 0.01 *	0.19 ± 0.02 *	0.74 ± 0.05 *
RJ600	50.13 ± 2.56 *	25.31 ± 3.20	0.87 ± 0.01 *	0.19 ± 0.08 *	0.75 ± 0.04 *
CQ	50.18 ± 2.06 *	23.13 ± 2.53 *	0.89 ± 0.45 *	0.19 ± 0.09 *	0.77 ± 0.01 *
NI	50.13 ± 1.78 *	23.01 ± 1.42 *	0.77 ± 0.13 *	0.16 ± 0.07 *	0.68 ± 0.03 *
NI400	50.31 ± 2.24 *	23.21 ± 0.87 *	0.75 ± 0.15 *	0.17 ± 0.01 *	0.66 ± 0.02 *

* Significant difference (*p* < 0.05) in the hepatic parameters of animals from other groups compared to NT.

**Table 3:** Mean values ± standard deviations of renal function parameters in test groups.

	Urea (mg/dL)	Creatinine (mg/dL)
NT	68.87 ± 5.24	3.47 ± 0.55
RJ200	41.13 ± 4.25 *	1.53 ± 0.16 *
RJ400	33.45 ± 4.24 *	1.21 ± 0.12 *
RJ600	33.63 ± 2.13 *	1.21 ± 0.08 *
CQ	33.24 ± 2.25 *	1.18 ± 0.21 *
NI	31.348 ± 1.87 *	1.29 ± 0.19 *
NI400	33.16 ± 2.84 *	1.27 ± 0.32 *

* Significant difference (*p* < 0.05) in the renal parameters of animals from other groups compared to NT.

**Table 4:** Mean values ± standard deviations of the hematological analysis (hemogram) in the test groups.

	Number of Erythrocytes (10^6^/µL)	Hematocrit (%)	Hemoglobin (g/dL)	Platelets (10^3^/µL)
NT	3.19 ± 0.83	28.89 ± 5.42	7.45 ± 1.98	320.24 ± 27.24
RJ200	4.89 ± 2.83	33.98 ± 10.35	11.24 ± 1.35	543.13 ± 89.13
RJ400	9.45 ± 0.89 *	43.13 ± 5.98 *	14.23 ± 1.93 *	730.55 ± 32.13 *
RJ600	9.55 ± 1.42 *	43.13 ± 1.46 *	14.45 ± 0.98 *	763.15 ± 29.23 *
CQ	9.79 ± 0.21 *	47.22 ± 0.53 *	14.98 ± 0.35 *	793.53 ± 24.88 *
NI	9.29 ± 0.83 *	45.76 ± 3.13 *	14.66 ± 0.13 *	734.13 ± 19.29 *
NI400	9.32 ± 0.99 *	48.13 ± 2.13 *	14.77 ± 0.01 *	766.75 ± 24.21 *

* Significant difference (*p* < 0.05) in the hemogram parameters of animals from other groups compared to the NT group.

**Table 5:** Mean values ± standard deviations of the hematological analysis (leukogram) in the test groups.

	Number of Leukocytes (10^3^/µL)	Lymphocytes (10^3^/µL)	Monocytes (10^3^/µL)	Segmented Neutrophils (10^3^/µL)
NT	130.24 ± 14.12	84.13 ± 6.29	4.13 ± 0.23	20.99 ± 7.13
RJ200	43.13 ± 13.83	33.12 ± 8.13	2.12 ± 2.21	14.13 ± 3.23
RJ400	5.24 ± 0.48 *	3.24 ± 0.13 *	0.29 ± 0.11 *	1.39 ± 0.47 *
RJ600	5.35 ± 0.97 *	3.21 ± 0.11 *	0.25 ± 0.27 *	1.49 ± 0.14 *
CQ	5.21 ± 0.84 *	3.11 ± 0.83 *	0.25 ± 0.03 *	1.52 ± 0.89 *
NI	5.42 ± 0.71 *	3.33 ± 0.28 *	0.34 ± 0.01 *.	1.74 ± 0.88 *
NI400	5.27 ± 0.21 *	3.46 ± 0.12 *	0.34 ± 0.01 *	1.71 ± 0.09 *

* Significant difference (*p* < 0.05) in the leukogram parameters of animals from other groups compared to the NT group.

## Data Availability

All of the data are present in the manuscript.
